# Supplementary material for: Isolation of a Divergent Strain of Bovine Parainfluenza Virus Type 3 (BPIV3) Infecting Cattle in China
Source: Viruses. 2019 May 29;11(6):489. doi: 10.3390/v11060489 (PMC6631270; doi:10.3390/v11060489)
Supplement: Supplementary file 1 [file viruses-11-00489-s001.pdf]

Table S1. Primers for detection of potential viral infectious agents of bovine respiratory disease complex (BRDC).

Virus	Primers	Sequence (5' - 3')
BPIV3	BPIV3-NF	TAGCTCTCCTGTTCTTATCTCAC
	BPIV3-NR	TGTCTAAATGCCTCTAATCG
BRSV	BRSV-F	TGCTATGTCTCGATTGG
	BRSV-F	CTTGAGATTAGCTCTAGCATC
BHV-1	BHV-gEf	ACGAGGAGACGCAGTTGGC
	BHV-gEr	TGCCTCGGGGTGGAAGAT
BVDV	BVDV-F	GGGNAGTCGTCARTGGTTCG
	BVDV-R	TGTGCCATGTACAGCAGAGYTT

Table S2. The primers used for RT-PCR amplification the complete genome sequences.

Primer	Sequence (5' - 3')
BPIV1F	5' ACCAAACAAGAGGAGAGACTTG 3'
BPIV1R	5' GTTGGTTGATTTTGGGTCG 3'
BPIV2F	5' CACATGAAGAAAATCAGTAGT 3'
BPIV2R	5' TCTGTTCCTCTCGATTGC 3'
BPIV3F	5' CATCCTGGAACATCCTCAAC 3'
BPIV3R	5' TAGACTATCATCTCCGTGGC 3'
BPIV4F	5' CTACCACTTGGGTTAGCTAGA 3'
BPIV4R	5' ACAATCAGATTACCTACAGAAC 3'
BPIV5F	5' ATAGGGATAGCCACCTCA 3'
BPIV5R	5' CCTTATTTCCTGCAACAT 3'
BPIV6F	5' TAGCAATGAACCCTATGTCCT 3'
BPIV6R	5' TGATGGGTTTAGTGGATA 3'
BPIV7F	5' ATGGACTATCCCAATGAGGC 3'
BPIV7R	5' ACCCATCTATTGTTTCCCAC 3'
BPIV8F	5' GGTATAATCCATTCAAGACA 3'
BPIV8R	5' GTATATGTCTGTTGACTTGAA 3'
BPIV9F	5' TATCGCACTAATGTATCCC 3'
BPIV9R	5' CTGAAGCCCAGTCTAAAA 3'
BPIV10F	5' ACCAGGTGAATCTTCGTTTCTAGAC 3'
BPIV10R	5' CTCCTTAAGATTGTCCCGGTCAAG 3'
BPIV11F	5' TAGTACATCACTTATCAGGGTAAGC 3'
BPIV11R	5' CTTACCTACTAGTGAGACTTCTG 3'
BPIV11R1	5' GGACCAAGTGTAGCATCATAAC 3'
BPIV12F	5' GCATGTTATGATGCTACACTTG 3'
BPIV12R	5' ACCAAACAAGAGAAAAACTCTGT 3'
